# Supplementary material for: Fine-scale genetic structure and rare variant frequencies
Source: PLoS One. 2024 Nov 5;19(11):e0313133. doi: 10.1371/journal.pone.0313133 (PMC11537391; doi:10.1371/journal.pone.0313133)
Supplement: S1 File — (DOCX) [file pone.0313133.s001.docx]

**Supporting information**

**Fine-scale genetic structure and rare variant frequencies**

Laurence Gagnon^1,2^, Claudia Moreau^1,2^, Catherine Laprise^1,2,3^, Simon L. Girard^1,2,4*^

**Table of contents**

[Supplementary figures 1](#_Toc177029917)

[S1 Fig. Pairwise sum of IBD segments length and number of IBD segments according to different genetic relatedness filters (pihat 0.125, pihat 0.25 and no cleaning). 1](#_Toc177029918)

[S2 Fig. PCA for each dataset of PFE. 2](#_Toc177029919)

[S3 Fig. UMAP clustering with DBScan for each dataset of PFE. 3](#_Toc177029920)

[S4 Fig. PCA of the final merge dataset. 4](#_Toc177029921)

[S5 Fig. UMAP colored according to the origin or ethnocultural group and shaped according to the clustering. 5](#_Toc177029922)

[S6 Fig. Heatmap of the averaged genetic relatedness (PLINK pihat) between and within clusters. 6](#_Toc177029923)

[Supplementary tables 7](#_Toc177029924)

[S1 Table. Populations and datasets. 7](#_Toc177029925)

[S2 Table. Number of individuals remaining according to different genetic relatedness filters. 8](#_Toc177029926)

[S3 Table. Table of the frequency and fold-increase of disease-causing variants known to be associated with a specific population. 9](#_Toc177029927)

[S4 Table. Mean, maximum and minimum proportion of pairs sharing an IBD segment through the genome for each PFE, clusters and reference population. 11](#_Toc177029928)

[Supplementary references 12](#_Toc177029929)

# Supplementary figures


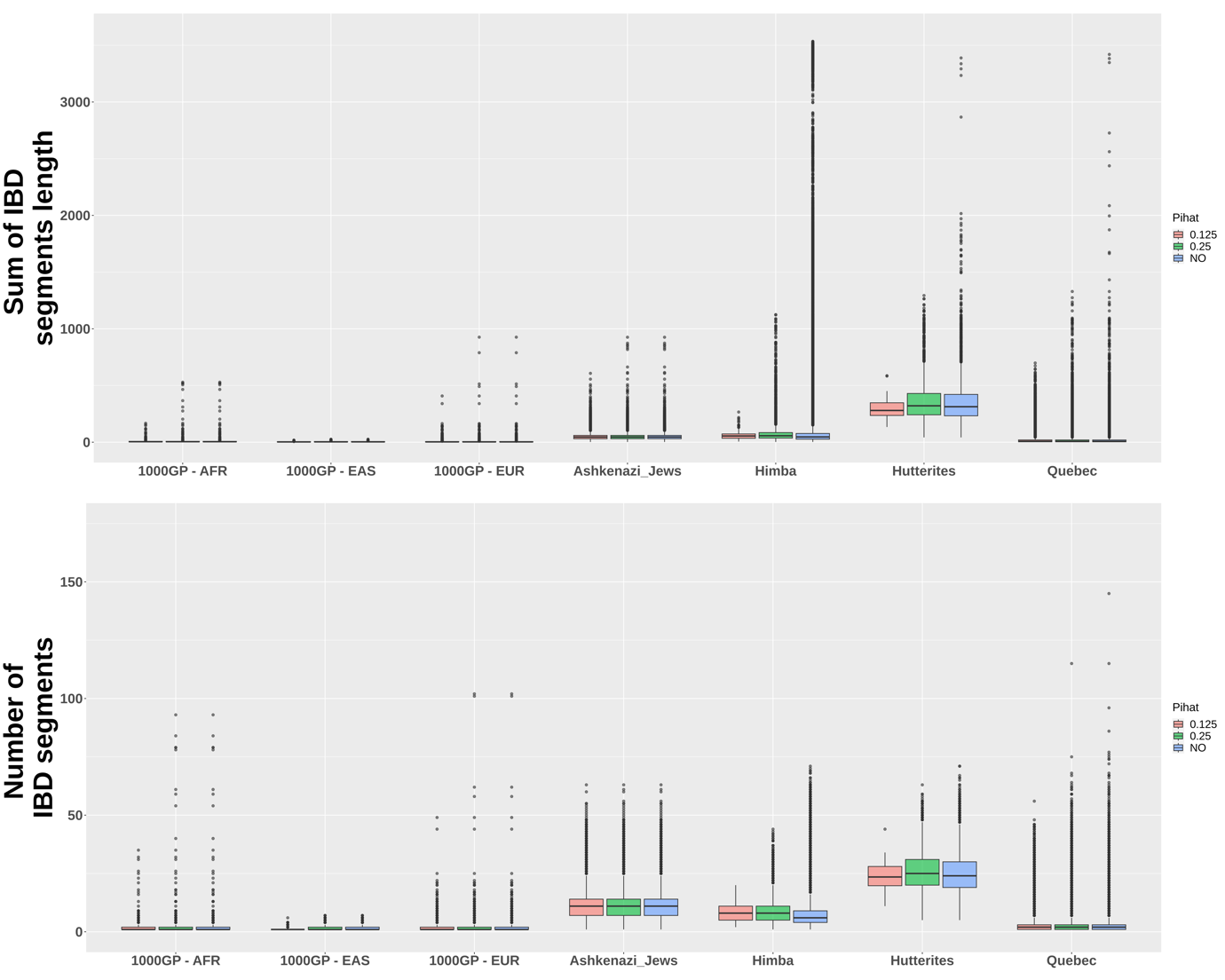


## S1 Fig. Pairwise sum of IBD segments length and number of IBD segments according to different genetic relatedness filters (pihat 0.125, pihat 0.25 and no cleaning).


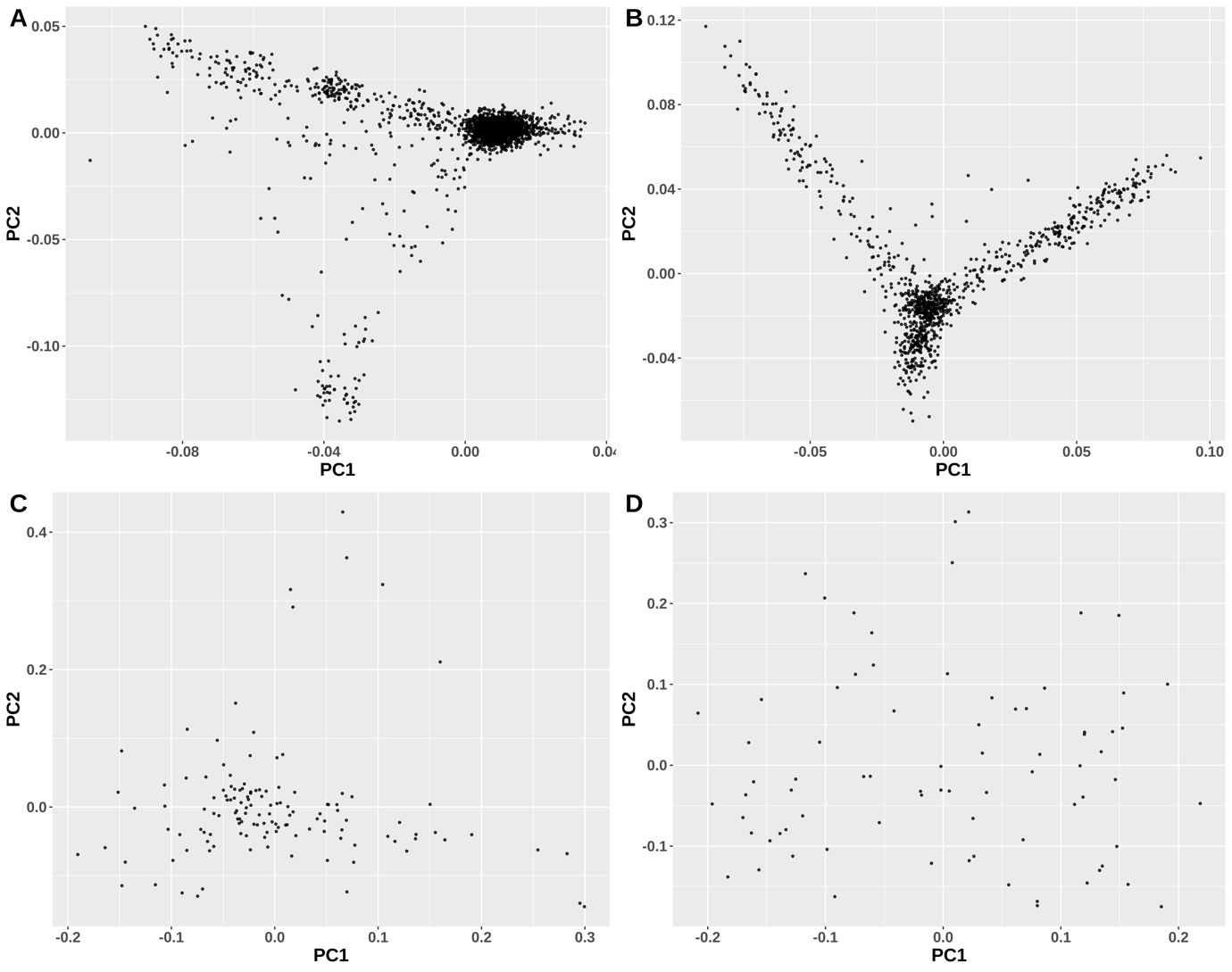


## S2 Fig. PCA for each dataset of PFE.

A. Ashkenazi Jews. B. Quebec. C. Himba. D. Hutterites


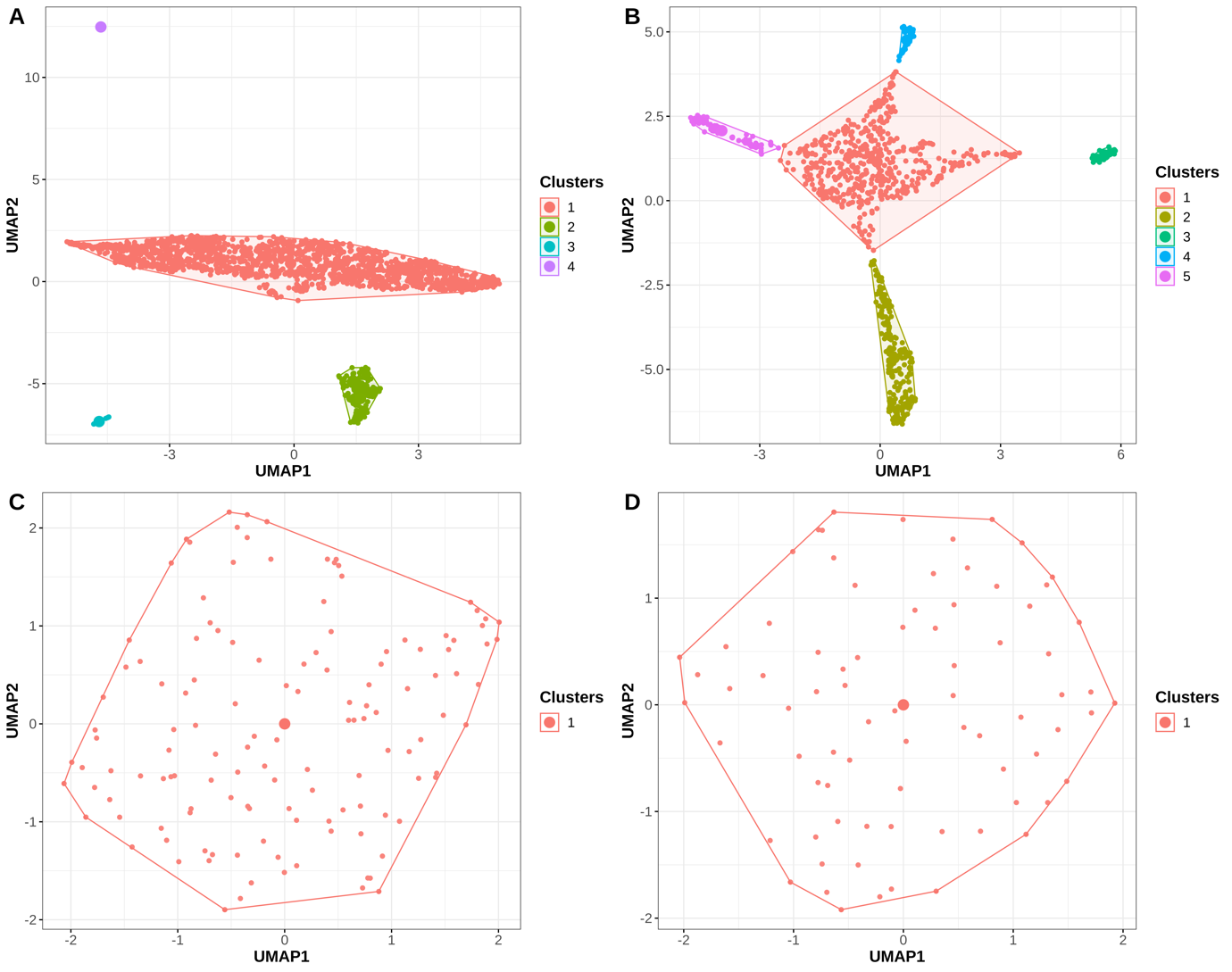


## S3 Fig. UMAP clustering with DBScan for each dataset of PFE.

A. Ashkenazi Jews. B. Quebec. C. Himba. D. Hutterites


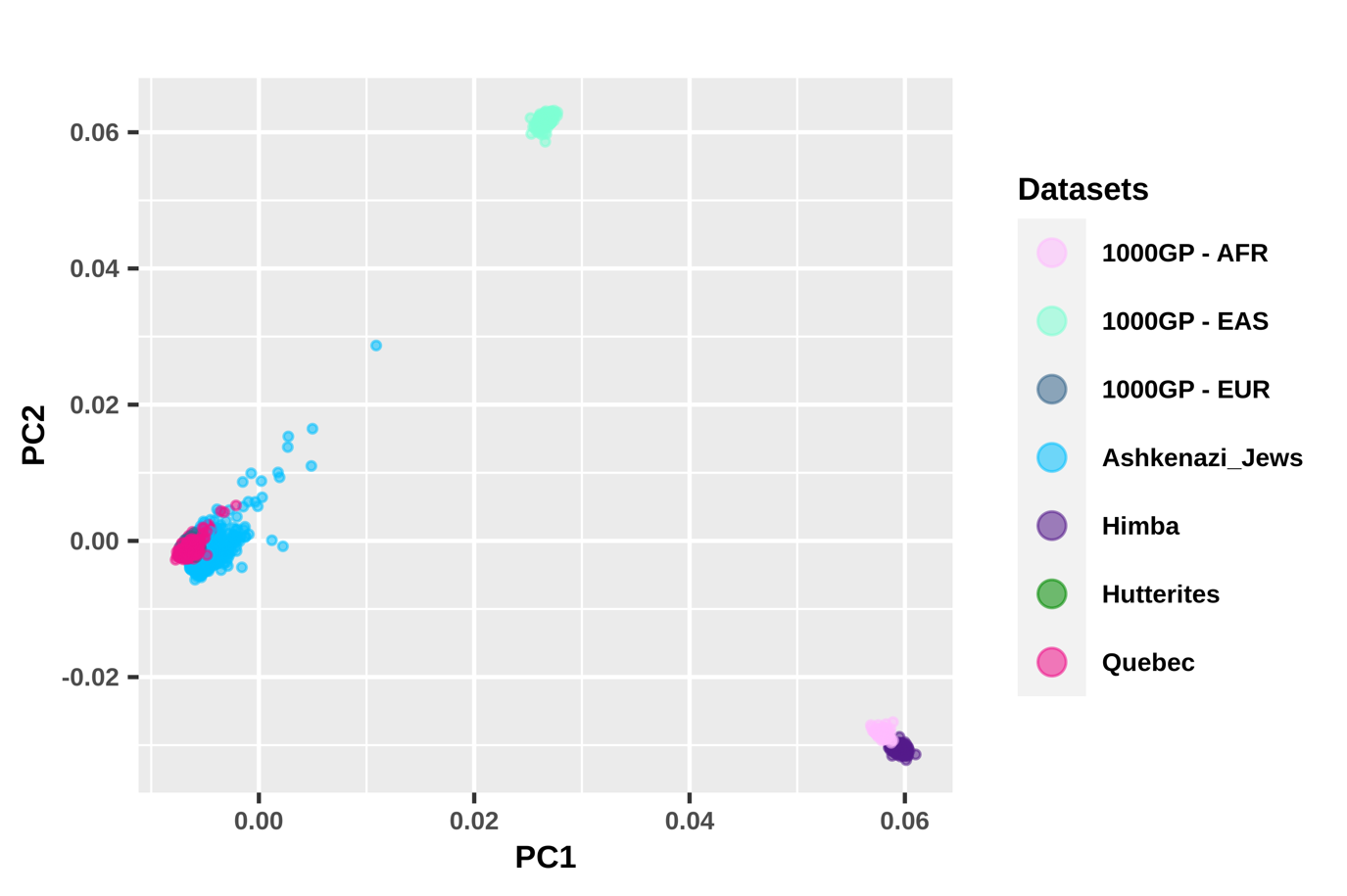


## S4 Fig. PCA of the final merge dataset.


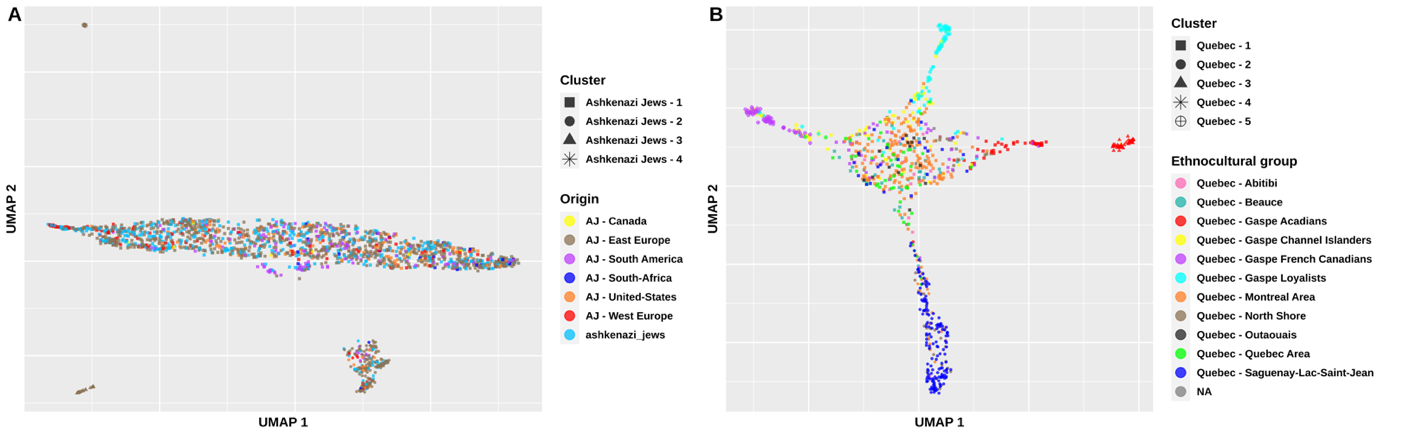


## S5 Fig. UMAP colored according to the origin or ethnocultural group and shaped according to the clustering.

A. Ashkenazi Jews. B. Quebec. For panel A, Ashkenazi Jews have reached these locations already long after the nearly complete mixing of the population in Eastern and Central Europe. Therefore, no genetic differences were expected across these locations (6).


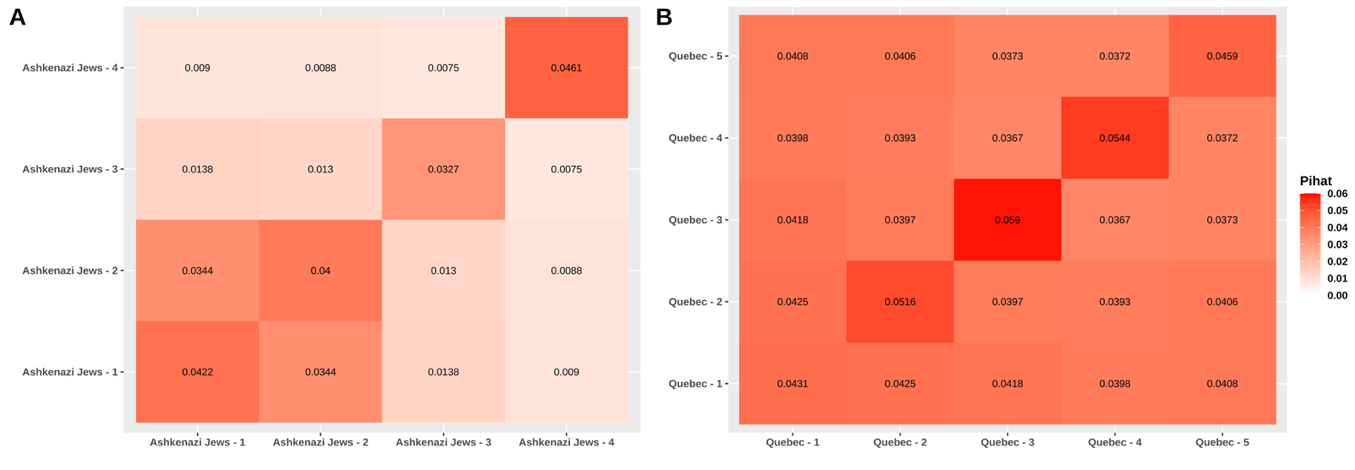


## S6 Fig. Heatmap of the averaged genetic relatedness (PLINK pihat) between and within clusters.

A. Ashkenazi Jews. B. Quebec.

# Supplementary tables

## S1 Table. Populations and datasets.

| **Cohorts** | **Sample size** | **Populations included** | **Genetic data** | **Sources** |
| --- | --- | --- | --- | --- |
| Quebec | 941 | French Canadian; English-speaking United Empire Loyalists and Acadians of the Gaspe Peninsula. | Illumina Omni Express or Illumina Omni 2.5 chips. | Quebec Regional Reference Sample [1] and unaffected individuals from the Saguenay-Lac-St-Jean asthma familial cohort [2]. |
| Ashkenazi Jews | 2,052 | Ashkenazi Jews. | Illumina HumanOmni1-Quad arrays. | dbGaP study accession number phs000448.v1.p1 [3]. |
| Himba | 131 | Himba. | Illumina MEGAex and H3Africa arrays. | dbGaP study accession number phs001995.v1.p1 [4]. |
| Hutterites | 77 | Hutterites. | SNP genotypes derived from sequence data. | dbGaP study accession number phs000185.v8.p1[5]. |
| African reference group | 85 | Mende of Sierra Leone (MSL). | SNP genotypes derived from sequence data. | 1000 Genomes Project [6]. |
| European reference group | 190 | British (GBR), Northern Europe and Western Europe (CEU). | SNP genotypes derived from sequence data. | 1000 Genomes Project [6]. |
| East Asian reference group | 207 | Han Chinese (CHB) and Japanese (JPT). | SNP genotypes derived from sequence data. | 1000 Genomes Project [6]. |

## S2 Table. Number of individuals remaining according to different genetic relatedness filters.

| **1000GP - AFR** | **1000GP - EAS** | **1000GP - EUR** | **Ashkenazi Jews** | **Himba** | **Hutterites** | **Quebec** | **Genetic relatedness** |
| --- | --- | --- | --- | --- | --- | --- | --- |
| 85 | 207 | 190 | 2052 | 675 | 98 | 952 | No filter |
| 85 | 207 | 190 | 2052 | 131 | 77 | 941 | 0.25 |
| 78 | 98 | 186 | 2044 | 27 | 9 | 880 | 0.125 |

## S3 Table. Table of the frequency and fold-increase of disease-causing variants known to be associated with a specific population.

Only the main population and the associated clusters are shown in the table.

| **Population**  **(count)** | **Cluster**  **(count)** | **Disease** | **SNPs** | **MAF of the European reference group of 1000GP** | **Mean MAF of 1000 resamplings (P-Value)** | **MAF of the associated cluster** |
| --- | --- | --- | --- | --- | --- | --- |
| Ashkenazi Jews (2052) | Ashkenazi Jews-1 (1729) | Gaucher disease (ClinVar: 4290) | chr1:155235843:T:C | 0.0026 | 0.0281 (<0.0001) | 0.0328 |
| Ashkenazi Jews (2052) | Ashkenazi Jews-1 (1729) | Maple syrup urine disease type 1B (ClinVar: 11937) | chr6:80168945:G:C | 0.0000 | 0.0015 (0.116) | 0.0018 |
| Ashkenazi Jews (2052) | Ashkenazi Jews-1 (1729) | Fanconi anemia (ClinVar: 12045) | chr9:95172033:T:A | 0.0000 | 0.0053 (0.063) | 0.0060 |
| Ashkenazi Jews (2052) | Ashkenazi Jews-1 (1729) | Usher syndrome type 1F (ClinVar: 4933) | chr10:54317414:G:A | 0.0000 | 0.0040 (0.128) | 0.0045 |
| Ashkenazi Jews (2052) | Ashkenazi Jews-1 (1729) | Niemann-Pick disease (ClinVar: 2980) | chr11:6394204:G:T | 0.0000 | 0.0020 (0.084) | 0.0024 |
| Ashkenazi Jews (2052) | Ashkenazi Jews-1 (1729) | ABCC8-related hyperinsulinism (ClinVar: 9088) | chr11:17397055:C:T | 0.0000 | 0.0047 (0.075) | 0.0054 |
| Ashkenazi Jews (2052) | Ashkenazi Jews-1 (1729) | Joubert syndrome type 2 (ClinVar: 197) | chr11:61393965:G:T | 0.0000 | 0.0062 (0.104) | 0.0068 |
| Ashkenazi Jews (2052) | Ashkenazi Jews-1 (1729) | Tay-Sachs disease (ClinVar: 3890) | chr15:72346234:C:G | 0.0000 | 0.0020 (0.383) | 0.0021 |
| Ashkenazi Jews (2052) | Ashkenazi Jews-1 (1729) | Canavan disease (ClinVar: 2605) | chr17:3499000:A:C | 0.0000 | 0.0063 (0.032) | 0.0071 |
| Ashkenazi Jews (2052) | Ashkenazi Jews-1 (1729) | Glycogen storage disease type 1A (ClinVar: 11998) | chr17:42903947:C:T | 0.0000 | 0.0055 (0.006) | 0.0066 |
| Quebec (941) | Quebec-2 (233) | Multiple intestinal atresia (Clinvar: 50608) | chr2:46994512:CAAGT:C | 0.0000 | 0.0016 (0.048) | 0.0043 |
| Quebec (941) | Quebec-2 (233) | Early-onset vitamin B6-dependent epilepsy (ClinVar: 503895) | chr8:37772802:GCAGA:G | 0.0000 | 0.0041 (<0.001) | 0.0151 |
| Quebec (941) | Quebec-2 (233) | Nephronophthisis type 2 (ClinVar: 660098) | chr9:100246788:G:A | 0.0000 | 0.0037 (<0.001) | 0.0129 |
| Quebec (941) | Quebec-2 (233) | Type 1A vitamin D hydroxylation-deficient rickets (ClinVar: 1664) | chr12:57766130:AC:A | 0.0000 | 0.0063 (<0.001) | 0.0172 |
| Quebec (941) | Quebec-2 (233) | Mucolipidosis II alpha/beta (CinVar: 2771) | chr12:101753469:TGA:T | 0.0000 | 0.0040 (<0.001) | 0.0151 |
| Quebec (941) | Quebec-2 (233) | Congenital disorder of glycosylation with hereditary phosphomannose isomerase deficiency (ClinVar: 14349) | chr15:74897050:G:A | 0.0000 | 0.0037 (<0.001) | 0.0129 |
| Quebec (941) | Quebec-2 (233) | Type I tyrosinemia (ClinVar: 11870) | chr15:80180230:G:A | 0.0026 | 0.0069 (<0.001) | 0.0259 |
| Quebec (941) | Quebec-2 (233) | Cystinosis (ClinVar: 4443) | chr17:3655305:G:A | 0.0000 | 0.0026 (0.006) | 0.0086 |

## S4 Table. Mean, maximum and minimum proportion of pairs sharing an IBD segment through the genome for each PFE, clusters and reference population.

The lines in grey indicate clusters with a mean IBD sharing higher than the entire population.

| **Population** | **Mean (%)** | **Maximum (%)** | **Minimum (%)** |
| --- | --- | --- | --- |
| Himba | 2.182 | 3.464 | 0.611 |
| Hutterites | 10.309 | 14.730 | 2.461 |
| Quebec | 0.396 | 0.819 | 0.073 |
| Quebec - 1 | 0.251 | 0.606 | 0.032 |
| Quebec - 2 | 2.130 | 3.630 | 0.359 |
| Quebec - 3 | 4.897 | 10.106 | 1.207 |
| Quebec - 4 | 3.013 | 8.078 | 0.595 |
| Quebec - 5 | 1.653 | 3.296 | 0.234 |
| Ashkenazi Jews | 1.211 | 1.770 | 0.081 |
| Ashkenazi Jews - 1 | 1.524 | 2.196 | 0.105 |
| Ashkenazi Jews - 2 | 0.261 | 0.633 | 0.012 |
| Ashkenazi Jews - 3 | 2.520 | 10.256 | 0.128 |
| Ashkenazi Jews - 4 | 5.986 | 27.895 | 0.526 |
| 1000GP - EUR | 0.094 | 1.470 | 0.006 |
| 1000GP - EAS | 0.048 | 1.351 | 0.005 |
| 1000GP - AFR | 0.171 | 1.261 | 0.028 |

# Supplementary references

1. Gagnon L, Moreau C, Laprise C, Vézina H, Girard SL. Deciphering the genetic structure of the Quebec founder population using genealogies. European Journal of Human Genetics. 2024 Jan 1;32(1):91–7.

2. Laprise C. The Saguenay-Lac-Saint-Jean asthma familial collection: the genetics of asthma in a young founder population. Genes Immun. 2014 Apr;15(4):247–55.

3. Lencz T, Guha S, Liu C, Rosenfeld J, Mukherjee S, DeRosse P, et al. Genome-wide association study implicates NDST3 in schizophrenia and bipolar disorder. Nat Commun. 2013 Nov 19;4(1):2739.

4. Scelza BA, Prall SP, Swinford N, Gopalan S, Atkinson EG, McElreath R, et al. High rate of extrapair paternity in a human population demonstrates diversity in human reproductive strategies. Science Advances. 2020 Feb 19;6(8):eaay6195.

5. Ober C, Nord AS, Thompson EE, Pan L, Tan Z, Cusanovich D, et al. Genome-wide association study of plasma lipoprotein(a) levels identifies multiple genes on chromosome 6q. J Lipid Res. 2009 May;50(5):798–806.

6. Auton A, Abecasis GR, Altshuler DM, Durbin RM, Abecasis GR, Bentley DR, et al. A global reference for human genetic variation. Nature. 2015 Oct;526(7571):68–74.
