# Supplementary material for: Fine-scale genetic structure and rare variant frequencies
Source: PLoS One. 2024 Nov 5;19(11):e0313133. doi: 10.1371/journal.pone.0313133 (PMC11537391; doi:10.1371/journal.pone.0313133)
Supplement: Attachment — Submitted filename: 2024_06_13_PONE-D-24-18509_Review.docx [file pone.0313133.s002.docx]

The primary objective of this study was to propose an approach on identifying fine-scale genetic structure of populations that can be used for discovering new rare genetic variants in association analyses. While the topic is interesting, the authors need to address several major concerns and include more detail on their method and analyses to strengthen their manuscript.

1. The authors did not discuss the current approaches, e.g., fineSTRUCTURE, ipPCA, etc., for identifying fine-scale population structure. It would be helpful to compare these approaches to their method, clearly state the novelty of their method, and apply the current approaches to the datasets explored in the manuscript to see whether fine structure can be captured within Ashkenazi Jews and the Quebec populations. The strengths/weaknesses of their method should be provided in the Discussion section.
2. In the Supplementary section, the authors stated that they used a threshold of “pihat >= 0.25” for filtering. Why did the authors choose to remove genetically-related individuals in their analysis?
3. Despite using a threshold of “pihat >=0.25”, it’s possible that the final dataset includes individuals with third-degree relatedness. Applying principal component analysis (PCA) to this is not appropriate as PCA is not robust to the presence of genetic relatedness, resulting in distortions in the overall PCA pattern. The authors need to use an adjusted PCA method, such as PC-AiR (Conomos et al., *Genet Epidemiol.*, 2015), which appropriately accounts for genetic relatedness.
4. The authors should provide a figure showing plots of the principal components (PCs).
5. The authors chose to use UMAP to visualize the population structure and labeled the points with the corresponding ethnicity and ancestry. As depicted in Figure 1 of the manuscript, UMAP tends to exaggerate the distinctiveness of populations, failing to show that genetic variation is a continuum. This issue was discussed recently in regard to the Nature paper, “Genomic data in the All of Us Research Program” ([see link for discussion](https://www.google.com/url?sa=t&source=web&rct=j&opi=89978449&url=https://www.nature.com/articles/d41586-024-00568-w&ved=2ahUKEwjc2727uNmGAxXT4MkDHTpSAzYQFnoECA4QAQ&usg=AOvVaw0jxLGb1wWqoNmovVl0h6gx)). Can the authors consider alternatives to UMAP for their approach? If not, the authors should at least state in the Discussion section the shortcomings of UMAP and their approach.
6. How did the authors decide on using the number of PCs for performing UMAP? Please provide more detail on this in the Results section.
7. Why did the authors choose specific populations within the African, East Asian, and European superpopulations for their analysis? In addition, the authors need to clearly define all population labels, i.e., AFR, EAS, EUR, etc., in Figure 1.
8. More detail should be provided on the DBScan method. Specifically, how did the authors tune the parameters, e.g., the epsilon value and “minPts parameter set to 4”, for this clustering approach? Did the authors consider other flexible clustering approaches that can “capture…clusters with various shapes” besides DBScan?
9. More detail needs to be provided on how the authors selected “known founder disease variants.” What method was used for the association analysis? Did the authors adjust for any confounders in their analysis? Given that the dataset includes genetically related individuals (third-degree relatedness), was this adjusted for in the association analysis?
10. In Table 1, the authors showed a p-value of 0, which is incorrect. Please provide the p-value in scientific notation. Furthermore, the authors should include the effect size and the 95% confidence intervals from their association analysis.
